# Family Changes and China’s Elderly Support System

**DOI:** 10.1093/geroni/igaa057.1950

**Published:** 2020-12-16

**Authors:** Zhan Hu, Ke Shen

**Affiliations:** Fudan University, Shanghai, China

## Abstract

Population aging has become the norm in China. Improving or reconstructing the elderly support system is therefore essential. While the government and institutional support has been improving and enhancing in China, family remains the most important source of elderly support for a long period in the future. Based on data from censuses and national surveys (1982-2015), we examine recent changes in household size and structure across ages from an individual life course perspective, to reveal the complexities and ambiguities behind the nationwide household change. Our findings suggest that multi-generational co-residence is a major vehicle to accommodate the needs of family members at critical stages of life. These salient features of family change in China call for new family-oriented policies, including pragmatic incentives to strengthen intergenerational solidarity, essential support for family caregivers, and diversified community services to care for frail elders.

